# A protocol for chronic pain outcome measurement enhancement by linking PROMIS-29 scale to legacy measures and improving chronic pain stratification

**DOI:** 10.1186/s12891-020-03696-2

**Published:** 2020-10-10

**Authors:** Patricia M. Herman, Maria O. Edelen, Anthony Rodriguez, Lara G. Hilton, Ron D. Hays

**Affiliations:** 1 RAND Corporation, Santa Monica, CA USA; 2 RAND Corporation, Boston, MA USA; 3 Government & Public Services, Deloitte Consulting, LLP, Los Angeles, CA USA; 4 Division of General Internal Medicine & Health Services Research, UCLA Department of Medicine, Los Angeles, CA USA

**Keywords:** Chronic pain, Chronic low back pain, PROMIS-29, Oswestry disability index, Roland-Morris disability questionnaire, High-impact chronic pain, Subgrouping, Linking, Crosswalks, Amazon mechanical Turk

## Abstract

**Background:**

Substantial investment has gone into research on the efficacy and effectiveness of pharmaceutical and nonpharmacologic interventions for chronic pain. However, synthesizing this extensive literature is challenging because of differences in the outcome measures used in studies of similar or competing interventions. The absence of a common metric makes it difficult to replicate findings, pool data from multiple studies, resolve conflicting conclusions, or reach consensus when interpreting findings.

**Methods:**

This study has a seven-member Advisory Council of chronic pain experts. Preliminary analyses will be performed on data from several large existing datasets; intermediate analyses will be performed using primary data collected from Amazon’s Mechanical Turk (MTurk); and cross-validation will use primary data collected from a nationally-representative, probability-based panel. Target sample size for both primary datasets is 1500. The three study aims are as follows:

- Aim 1 will develop and evaluate links between the 29-item Patient-Reported Outcomes Measurement Information System (PROMIS®-29) and legacy measures used for chronic pain such as the Roland-Morris Disability Questionnaire (RMDQ) and the Oswestry Disability Index (ODI). We will assess the best method of score linking and create crosswalk tables.
- Aim 2 will evaluate and refine the Impact Stratification Score (ISS) based on 9 PROMIS-29 items and proposed by the NIH Research Task Force on chronic low back pain. We will evaluate the ISS in terms of other indicators of condition severity and patient prognosis and outcomes and identify cut-points to stratify chronic pain patients into subgroups.
- Aim 3 will evaluate the strengths and limitations of MTurk as a data collection platform for estimating chronic pain by comparing its data to other data sources.

**Discussion:**

The accomplishment of Aims 1 and 2 will allow direct comparison of results across past and future studies of chronic pain. These comparisons will help us to understand different results from seemingly similar studies, and to determine the relative effectiveness of all pharmaceutical and nonpharmacologic interventions for chronic pain across different trials. Aim 3 findings will provide valuable information to researchers about the pros and cons of using the MTurk platform for research-based data collection.

**Trial registration:**

ClinicalTrials.gov: NCT04426812; June 10, 2020.

## Background

Substantial research has gone into determining the efficacy and effectiveness of pharmaceutical and nonpharmacologic interventions for chronic pain. Pharmaceutical interventions are still most commonly used [1], but a number of nonpharmacologic approaches have now been shown to be efficacious and effective, especially for chronic low back pain (CLBP), and included in guidelines [2–10]. While there is an extensive literature on these interventions for chronic pain, it is challenging to synthesize the findings because of differences in the samples and outcome measures used. The National Institutes of Health (NIH) Pain Consortium’s Research Task Force (RTF) on CLBP noted that these differences make it “difficult to compare epidemiologic data and studies of similar or competing interventions, replicate findings, pool data from multiple studies, resolve conflicting conclusions, develop multidisciplinary consensus, or even achieve consensus within a discipline regarding interpretation of findings” [11],^p1129^. These differences also limit answers to questions such as ‘Which therapies work best? And for whom?’

The lack of common outcome measures and the inability to identify meaningful subgroups of patients prompted the NIH RTF on CLBP to recommend the use of a common minimum data set and a scheme to classify CLBP patients by its impact on their lives [11]. The RTF recommended use of items in the Patient-Reported Outcomes Measurement Information System (PROMIS®)-29 for studies of CLBP but they also agreed that investigators could substitute “legacy” measures (commonly used measures) such as the Roland-Morris Disability Questionnaire (RMDQ [12]) if they preferred. Therefore, studies will likely continue to use a variety of outcome measures.

A number of crosswalks and links between PROMIS and legacy measures have been produced [13, 14] that enable an outcome score on one measure to be translated into a score on another measure. However, crosswalks have not yet been developed for some of the most commonly used measures for CLBP such as the RMDQ and the Oswestry Disability Index (ODI) [15]. In addition to enabling side-by-side comparisons among studies that used different measures, these crosswalks aid in the interpretation of the results of meta-analyses, and enable the harmonization required for detailed individual patient data (IPD) meta-analyses [16, 17]. Aim 1 of this study is to create empirical links between several common measures used in chronic pain studies and the PROMIS-29 to enable comparisons across studies.

The RTF also recommended that subgroups of CLBP patients be identified by stratifying them according to the impact CLBP has had on their lives. The US National Pain Strategy (NPS) has placed a focus on identifying those with high-impact chronic pain [18] and the NPS’s Population Research work group is considering measures of chronic pain impact [19]. Several measures have been used to classify or stratify patients by the impact of their chronic pain [11, 20–23]. The most well-studied of these is the classification scheme based on the 7-item Graded Chronic Pain Scale [21, 24–30]. Those with high-impact versus milder levels of chronic pain on this scale were found to have significantly greater healthcare utilization and higher healthcare costs [19, 21, 25, 31, 32]; worse health-related quality of life [21, 28, 31]; more unemployment and absenteeism [21, 31]; and more opioid use [21, 31, 32]. There is substantial variation across studies in baseline chronic pain impact levels [31]. This variation severely limits comparing the effectiveness of the interventions across studies because any differences could be attributed to variation in patient case-mix at baseline. Pain impact classification enables case-mix adjustment [33] or weighting, and subgroup analyses using methods such as IPD meta-analysis or simulation modeling. Moreover, targeting patients at the same chronic pain impact level would enhance trial efficiency by reducing patient heterogeneity, and researchers could later report on heterogeneity of treatment effect (HTE) using these groupings [34] allowing interventions to be directed at subgroups where they will be the most effective. The RTF’s impact Stratification Score (ISS) is based on 9 of the PROMIS-29 items identified based on analyses of a sample of 218 patients with LBP who received epidural steroid injections [11]. The results showed that the ISS was highly correlated (Spearman correlation) with the RMDQ (0.66) and ODI (0.81) at baseline, and more responsive to changes in symptoms than the RMDQ. The RTF went on to say that “further assessment of the reliability, validity, and clinical utility of this stratification strategy is a high priority.” [11], ^p1137^ But we are aware of only one study to further evaluate this stratification scheme [35]. Aim 2 of this study is to evaluate and refine the ISS to ensure that it creates meaningful impact-based sub-classifications of chronic pain patients.

This study will use data from three sources. Initial analyses will be performed using large existing datasets that contain the PROMIS-29 plus other measures administered to chronic pain patients. Intermediate analyses will be performed using primary data collected from Amazon’s Mechanical Turk (MTurk), and cross-validation will use primary data collected from KnowledgePanel, a nationally representative probability-based sample [36].

MTurk is the most commonly researched crowdsourcing platform in science and it employs over 500,000 participants [37]. Numerous published studies of data collection using the MTurk platform exist in the social science disciplines [38], and there is an emerging literature on its utility for research on clinical populations [39–41]. One of the key reasons MTurk is attractive to researchers is the opportunity for rapid and inexpensive data collection. For example, data collection for small samples can be completed within a few hours, and the payment for research-related tasks is typically set at the federal minimum wage [42]. In addition, MTurk was found to be less costly and yielded higher quality data than samples recruited from Facebook, Google AdWords, or Craigslist [43]. Further evaluation of this efficient data collection method to verify data quality and improve its application could dramatically reduce the cost of future chronic pain research. Aim 3 of this study is to evaluate MTurk as a data collection method in terms of cost, time to complete, data quality, response at follow-up, relationships among variables, and sample representativeness.

In summary, this study consists of two chronic pain measure improvement efforts (crosswalk/links between measures and further development of an impact stratification scheme) and evaluation of an efficient data collection platform (MTurk). These are the specific aims of this study:

- Specific Aim 1: Develop and evaluate links or crosswalks between the PROMIS-29 and other common (legacy) measures used for chronic pain so that the results of studies using different measures can be compared.
- Specific Aim 2: Evaluate and refine the RTF proposed chronic pain impact stratification scheme that is based on 9 PROMIS-29 items.
- Specific Aim 3: Evaluate MTurk as a cost- and time-efficient method to collect quality data on individuals with chronic pain.

Note that although CLBP is a main focus in this study (i.e., our named measures to link to PROMIS are CLBP measures and it was the NIH RTF on CLBP that developed the ISS), dataset availability and our Advisory Council’s advice may allow us to link PROMIS to measures for other types of chronic pain and to test the ISS in other chronic pain populations [35].

## Methods/design

**Fig. 1:**
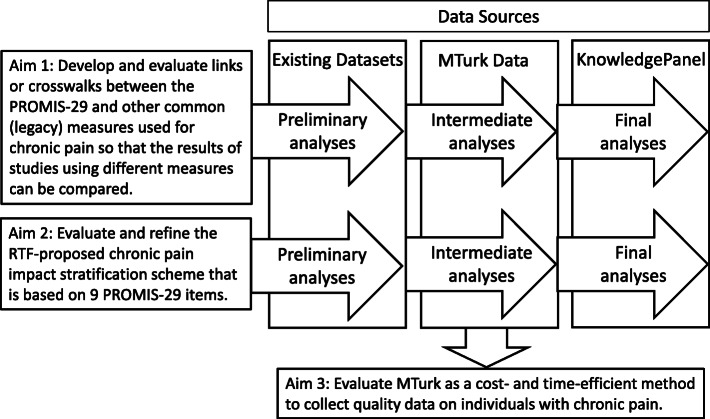
Overview of Study. Legend: KnowledgePanel = a nationally representative probability-based online survey panel from which we will gather data from a representative sample of chronic pain patients; MTurk = Amazon’s Mechanical Turk, an crowdsourcing platform through which we will gather data from a convenience sample of chronic pain patients; PROMIS-29 = 29-item Patient-Reported Outcomes Measurement Information System; RTF = National Institutes of Health Pain Consortium’s Research Task Force on Chronic Low Back Pain. Aims 1 and 2 use data from three sources for their preliminary, intermediate and final analyses. The first source is made up of available existing data and the last two sources will involve primary data collection. Aim 3 involves additional analyses to establish the usefulness of MTurk as a platform for data collection

This study will be performed by researchers at the RAND Corporation and the University of California Los Angeles (UCLA) with input and advice from a seven-member Advisory Council of experts in chronic pain and its measurement and in data collection. The study will use at least three large existing datasets for the initial analyses for Aims 1 and 2. Further analyses will use data collected via MTurk and these results will be cross-validated using data from KnowledgePanel, a nationally-representative probability-based sample. These data collection efforts have not yet begun. Figure 1 shows the overall plan for the study.

The RAND Human Subjects Protection Committee has reviewed and approved this study as exempt (2019–0651-AM02).

### Advisory council

The Advisory Council will meet about twice a year and provide input on priorities, suggest existing datasets we could use and legacy measures for which linking to PROMIS-29 are most important. Members of the Council have also agreed to be available as needed to provide input and answer questions as the study progresses.

### Existing datasets

We have three existing datasets from RAND studies that contain measures of interest and have large enough samples for initial analyses (Table 1). These were collected in the RAND Center of Excellence for the Appropriateness of Care (CERC) study [44–46], Assessment of Chiropractic Treatment for Low Back Pain (ACT) trial [47, 48], and Crowdsourcing for Patient Engagement (MTurk) study [49]. We will use these to begin to create links or crosswalks of the PROMIS-29 scales with the ODI (the CERC and existing MTurk datasets) and the RMDQ (the ACT dataset) and explore crosswalks/links of the PROMIS-29 scales with the Neck Disability Index (the chronic neck pain, or CNP, portion of the CERC dataset). Also, because these datasets include the PROMIS-29, they also include the 9 items that make up the RTF-proposed ISS and can be used for analyses of that measure. These datasets also contain measures of various aspects of individuals’ lives where chronic pain can have an impact—e.g., healthcare utilization, work status, and mood—allowing cut points to be explored.

**Table 1:** In-house existing datasets that will be used in initial studies

	CERC	ACT	MTurk	
Sample size	*n* = 2024 (*n* = 518 CLBP only; *n* = 347 CNP only; *n* = 1159 both)	*n* = 750 (*n* = 384 CLBP; *n* = 287 acute LBP; *n* = 79 subacute)	*n* = 5755	*n* = 1444
Population	Chiropractic patients being treated for CLBP and/or CNP	Active military with LBP participating in RCTs on chiropractic	General population	Those with CLBP
Longitudinal?	Yes	Yes	No	No
Average age in years (SD)	48.6 (14.5)	30.9 (8.7)	36.2 (10.9)	37.7 (11.2)
% Female	72.4%	23.3%	52.4%	56.4%
Average pain 0–10 (SD)	3.1 (1.8)	4.6 (2.0)	2.2 (2.3)	3.9 (2.2)
Measures used:				
PROMIS-29	Yes	Yes	Yes	Yes
ODI	Yes	No	No	Yes
RMDQ	No	Yes	No	No
NDI	Yes	No	No	No
Pain NRS 0–10	Yes	Yes	Yes	Yes

*ACT* Assessment of Chiropractic Treatment for Low Back Pain, Military Readiness and Smoking Cessation, *CERC* RAND Center of Excellence for the Study of Appropriateness of Care in CAM: *CLBP* chronic low back pain, *CNP* chronic neck pain, *MTurk* data collected using an Amazon Web Service called Mechanical Turk, *ODI* Oswestry Disability Index, *PROMIS-29* short form of the Patient-Reported Outcomes Measurement Information System, *RMDQ* Roland-Morris Disability Questionnaire, *NRS* = numerical rating scale

In the first year of the study we will use our Advisory Council and other sources to identify other existing datasets which could be mined for this study. Other promising existing datasets include the PROMIS Wave 1 data [50], the American Chronic Pain Association Supplement from 2007 [51], and the PROMIS Profiles-HUI data [52].

### Additional primary data collected via MTurk

Starting in Year 2 of this study the MTurk platform will be used to collect additional data in support of the work involved in Aims 1 and 2. These data will include items or measures not captured (or not captured together) in the existing datasets (e.g., ODI and RMDQ together, the Graded Chronic Pain Scale), and data from targeted subpopulations missing from or not sufficiently represented in the existing datasets (e.g., individuals with more severe CLBP or other types of chronic pain). The specifics of the inclusion and exclusion criteria for the MTurk samples and the items included will be determined through the Year 1 and early Year 2 work using the existing datasets, as well as recommendations from the Advisory Council. Given our past experience recruitment for this survey should take approximately 1 month. In order to assess responsiveness to change for Aim 2, all participants in the initial survey will also be asked to complete a shorter follow-up survey 3 months later.

### Knowledge panel

The KnowledgePanel [36] is a nationally representative online survey panel originally developed by GfK and now owned and maintained by Ipsos Public Affairs. In Year 3 it will be used to cross-validate all study results generated from the other data sources. This panel has more than 55,000 individuals recruited through a probability-based sampling methodology (address-based sampling) that improves population coverage for hard-to-reach individuals and computers and internet access are provided for those who do not have them. Given the large size of the underlying panel, sample sizes for the KnowledgePanel could go as high as 2000 completed surveys of patients with CLBP. The existing panel also allows completed data collection within 2–3 weeks of fielding the survey. In order to assess responsiveness to change for Aim 2, and similar to our plans for the MTurk data, all panelists responding to the initial survey will also be asked to complete a shorter follow-up survey 3 months later.

### Approach to aim 1: links and crosswalks between PROMIS and legacy chronic pain measures

The main effort in this study will be to link elements of the PROMIS-29 to the ODI and the RMDQ, the two most common patient-reported outcomes (PROs) [53] used in studies of CLBP, the most common type of chronic pain [54, 55]. According to one systematic review of CLBP studies, out of 354 randomized trials published between 2001 and 2010, 168 (47%) used the ODI and 132 (37%) used the RMDQ [53]. Thus, the discussion below focuses on the ODI and RMDQ. However, our existing datasets (see Table 1) will also allow us to explore linkages of PROMIS-29 scales to the Neck Disability Index (NDI) [56], the most common PRO used for CNP [57], which is the second most common type of chronic pain [55]. Although below we only discuss our plans for the RMDQ and ODI, our goal will be to use similar methods to also create crosswalks/links between at least two other commonly used PROs and the PROMIS-29.

We will link PROMIS-29 physical function, pain interference and pain intensity scores with the ODI and RMDQ. First, we will assess whether equating, scale alignment, or prediction is appropriate for each pair of measures [58]. We will evaluate whether the PROMIS-29 scales, ODI, and RMDQ measure the same underlying concept (i.e., are “sufficiently” unidimensional to calibrate items on the same metric) using categorical confirmatory factor analytic models for all items in pairs of instruments. For example, we will estimate models that include the 4 PROMIS-29 physical functioning items, the 4 PROMIS-29 pain interference items, the PROMIS-29 pain intensity item, and the 24 RMDQ items. We will estimate one-factor models and evaluate model fit using the comparative fit index (CFI), root mean square error of approximation (RMSEA), and standardized root means square residual (SRMR). CFI values of 0.95 or above, RMSEA values of 0.06 or below, and SRMR values of 0.08 or below will provide support for model fit [59]. We are also mindful of simulation work that shows that fit criteria can be affected by the number of items and the distribution of the data [60, 61]. We will also inspect model modification indices to help determine if modification to the model or a subset of items provide better fit to the data. In addition, we will evaluate the assumption of local independence by examining residual correlations among items; residual correlations of 0.20 or above are indicative of potential violation of local independence [62].

For sets of items in the pairs of instruments that are sufficiently unidimensional, we will fit the item response theory (IRT) graded response model [63] to estimate item parameters (thresholds, discrimination) on the same underlying metric. Given their popularity in a segment of the IRT community, we will also evaluate the relative suitability of the more parsimonious family of Rasch models for graded data (e.g., partial credit model) [64] in parallel to the graded response model by constraining discrimination parameters to be equal (i.e., estimate thresholds only). If we observe sufficiently high correlations among scales (e.g., 0.80) and suitable model fit to items from pairs of scales for either IRT modeling approach, we will use item parameter estimates from fixed-parameter calibration to construct a crosswalk table using expected a posteriori (EAP) summed scoring and crosswalk tables that map raw summed scores from the ODI and RMDQ to the PROMIS-29 physical function, pain interference and pain intensity scores [14, 65]. Although preliminary studies suggest that correlations between entire scales will not be high enough to support this approach for all linking, we will explore the possibility of identifying subsets of items from disparate measures that can be cross-walked in this way.

If the correlations of PROMIS-29 scales and legacy measures are not large enough (0.80 or higher) to support the methods discussed above, we will develop models to predict PROMIS-29 scale scores from the ODI and RMDQ (and vice versa). We will entertain a variety of a models including ordinary least squares regression, the limited dependent variable mixture model, the beta-based regression approach, and Bayesian models. In addition, we will compare estimated scores with observed scores overall and by patient characteristics (e.g., age, gender, duration of low back pain). We will also estimate mean-error and root-mean-squared error for different models. During prediction, we will account for regression to the mean [66]. We will also evaluate whether the prediction equation varies by patient characteristics (age, gender, duration of LBP, etc.) and over time.

We will compare estimated PROMIS scores from the ODI and RMDQ with observed scores within the sample used to derive them and in independent samples (i.e., other existing datasets, new data via MTurk and the KnowledgePanel) [67]. We will evaluate the accuracy of equating and predictions at the group and individual levels. These estimates will be used to provide guidance on the uncertainty in estimates of one measure from other measures in group-level (e.g., research) studies. Based on previous work, we expect that these estimates will also indicate caution in using crosswalks or other links for individuals because of the relatively larger errors. We will provide confidence intervals around estimated scores from one measure to another.

### Approach to aim 2: evaluate and refine an impact subclassification scheme for chronic pain

In 2014 the NIH RTF recommended stratifying CLBP by its impact on patients’ lives according to pain intensity, pain interference with normal activities, and functional status [11]. The RTF’s proposed ISS sums the raw scores from 9 of the PROMIS-29 items covering physical function, pain interference, and pain intensity. The result is a total score with a possible range from 8 (least impact) to 50 (greatest impact) [11]. The NIH RTF allocated approximately equal percentages of individuals into three categories for the ISS: 8–27 (mild), 28–34 (moderate), and 35–50 (severe). We will estimate the percentages in these three ISS categories in each of our datasets. In addition, we will estimate internal consistency reliability and construct validity using Spearman rank-order and product-moment correlations between the ISS and variables with which it is hypothesized to be associated—e.g., healthcare utilization, worker productivity, and mood (depression and anxiety). We will also examine the correlations of the ISS with the ODI and RMDQ.

Further, we will evaluate responsiveness to change in the ACT dataset, MTurk and KnowledgePanel data using ANCOVA with the ISS as the dependent variable and a retrospective rating of change (Compared to 3 months ago, your low back pain is: *much worse, a little worse, about the same*, *a little better*, *moderately better, much better* and *completely gone*) as the independent variable. We will identify which ISS items are most responsive to change and use those reporting “about the same” to estimate test-retest reliability. The components that make up the ISS may differ by individual characteristics such as demographics and length of time patients have had chronic pain. We will examine whether low ISS scores are driven by pain intensity and higher scores by interference and/or physical function (similar to the structure of the Graded Chronic Pain Scale [21]), or if individuals’ scores or ISS categories are being driven mostly by (or by some particular pattern across) pain intensity, pain interference, or physical function. We will compare the grades of chronic pain based on the most well-studied and validated impact stratification scheme, the Graded Chronic Pain Scale [21, 24–30], to the range of ISS to see what this comparison offers in terms of appropriate ISS cut-points.

We will examine the ISS as an independent variable with the retrospective rating of change item as a dependent variable in an area under the curve analysis [68]. The retrospective rating of change item will be coded as improved for people reporting they are moderately better, much better, or pain is completely gone; all other categories will be coded as not improved. Finally, we will examine other dichotomizations of the item to assess the robustness of the estimates.

### Approach for aim 3: evaluate MTurk as a cost- and time-efficient data collection method

Starting in Year 2, the MTurk platform will be used to collect data in support of the work conducted in Aims 1 and 2. The existing datasets contain the key measures of interest and are large enough to power initial analyses. However, they do not include the broad array of measures that might be useful, and none were intended to be representative of all CLBP patients. For example, the CERC dataset only includes CLBP and CNP patients currently being treated by a chiropractor, and the ACT sample only includes Active Duty military who were chiropractic clinical trial participants. The addition of MTurk data will be used to validate the relationships estimated on the existing datasets to see whether these estimates hold up in other samples, and allow additional measures to be included and tested for relevance before we move to the nationally representative KnowledgePanel sample for final evaluation. The KnowledgePanel data collection will replicate measures and participant characteristics captured in the new MTurk data. Therefore, we can evaluate the utility of the MTurk platform by comparing those data to the nationally representative sample from KnowledgePanel.

The new MTurk data collection will include chronic pain measures not previously concurrently collected in the other existing datasets (e.g., both the ODI and RMDQ) and will capture these measures in broader chronic pain populations. For example, a number of studies now indicate the importance of patient catastrophizing on chronic pain outcomes [69–72], and that the benefits of certain therapies are affected by changes in catastrophizing [73–77]. Catastrophizing is included in the CERC and existing MTurk datasets, however, a more detailed analysis of its effects related to the RMDQ might be desired. Also, the Graded Chronic Pain Scale [21] is not included in our existing datasets and given its extensive use it would be good to compare its grades to the ISS scores seen. Finally, patients’ retrospective rating of change, used in test-retest reliability and the sensitivity of measures to change, is captured in the ACT data, but not in the CERC or existing MTurk data.

The MTurk platform provides the opportunity to draw samples from different subgroups with different chronic pain conditions, using any set of items and over any time interval. The process for obtaining survey data via the MTurk platform is as follows: The MTurk surveys will be designed using SelectSurvey and then posted on the MTurk platform. MTurk participants who consent will first be administered a survey eliciting demographic information, their health conditions from a general health checklist, and the PROMIS-29. All respondents will receive $1 for this initial health screening survey. MTurk participants who endorse CLBP will be invited to answer additional questions including the ODI, RMDQ, and/or any other legacy measures of interest, for a “bonus” of $1.50. We will also employ MTurk to collect longitudinal data, which has been shown to be feasible in other studies (pilot data had an 80% response rate for second round data) [78]. MTurk participants are anonymous, but an intermediary platform (TurkPrime) allows the inclusion or exclusion of previous participants in new surveys. This can be done through various options including limiting survey responses to single respondents, or by sending emails to anonymous MTurk participants via the platform [79]. We plan to recruit participants for longitudinal data collection by emailing them survey links through their MTurk accounts using unique MTurk Worker IDs. They can view the survey announcement and participate if they choose. Respondents remain anonymous throughout this process.

In this study we will measure data quality, leveraging lessons from previous studies [80]. For example, to increase truthfulness of the anonymous respondents, we employ a two-tiered survey process by posting the surveys as “Brief Health Surveys” and piping respondents over to the full survey with bonus only if they endorse the conditions of interest. This reduces the likelihood of respondents simply endorsing CLBP to get paid to answer a survey on that condition [81]. To reduce selection bias, we will deploy small batches of surveys hourly throughout a several-week time period. This ensures sampling from individuals who are online at different times throughout the day and available on different days. We will implement attention checks to ensure the respondents are people and not robots, and to ensure respondents are paying attention. We will especially track meta-data such as time to complete each question and the survey, and missing data for the key measures of interest (PROMIS, ODI, RMDQ, and other legacy measures). Finally, MTurk participant user forums (i.e., Turkopticon) will be monitored for potential chatter related to the study.

In addition to providing a rich dataset to enhance the analyses performed for Aims 1 and 2 on existing data, we will compare the MTurk data to that gathered through KnowledgePanel. We will compare the cost and time to complete surveys, data quality, response at follow-up, relationships (correlations) seen between key variables, and sample representativeness in terms of demographics, duration of pain, proportions with different levels of the ISS, and PROMIS-29 scale scores. For data quality, we will include the same attention checks in the KnowledgePanel survey as are included in the MTurk survey and compare percentage of failures across datasets using χ^2^. The response rate at 3-month follow-up will also be compared using χ^2^. We will calculate and compare Spearman rank-order and product-moment correlations between key variables across data sources, and we will compare outcome effect sizes. Student’s t-tests will be used for continuous outcome and demographic variables. Effect sizes for each parametric test will be calculated with Cohen’s *d*. Chi-square tests of independence will be used with nominal independent variables, non-scalar dependent variables of categorical outcomes, and demographics variables. Chi-square measures will be used to assess association and effect sizes will be calculated with Cramer’s V to indicate the strength of association. Based on this information we will make recommendations for data collection using MTurk, including best practices, cautions, and the situations where it would be appropriate to use.

### Sample sizes

For confirmatory factor analysis, rules of thumb have been offered about the minimum number of subjects per each parameter to be estimated (e.g., at least 10 subjects per parameter [82]). If a measure is to be used in a specific subgroup (e.g., those with longer duration of pain), then a sufficient sample size is needed to represent that subgroup. It has been suggested that sample sizes of 200 are needed for the Rasch model for dichotomous items [83]. At this sample size, SEs of item thresholds are in the range of 0.14 to 0.21 (based on [2/(square root of n)] < SE < [3/(square root of n)], where n is the sample size). For graded-response models, a sample size of 500 is recommended [83]. Although at least 500 is desirable, a smaller sample could still provide useful information, depending on the properties and composition of the scale. In general, the ideal situation is to have adequate representation of respondents for each combination of possible response patterns across a set of items—something that is rarely achieved. It is important, however, to have at least some people respond to each of the categories of every item. We will have data with large numbers of CLBP patients from three existing studies available with wide distributions of item responses that will make possible the proposed initial analyses. The standard errors of correlations are 0.07, 0.06 and 0.05 for sample sizes of 200, 300 and 400, respectively. Logistic and other types of regression equations have lower sample size requirements. To ensure that we have sufficient power for subgroup analyses, sufficient representation of all possible response patterns, and a nationally representative sample sufficient for validation of all results, our MTurk data collection and KnowledgePanel sample will aim for sample sizes of 1500 each.

## Discussion

The intent of Aims 1 and 2 is to allow direct comparison of results across past and future studies of CLBP. These comparisons will help us to understand why similar studies yield different results, and to determine the relative effectiveness of all pharmaceutical and nonpharmacologic interventions for chronic pain, even if they were not directly compared in a trial.

In particular, the results of Aim 1 will enable side-by-side comparisons among studies using different measures. In addition, these links and crosswalks will aid in the interpretation of the results of meta-analyses, and enable the harmonization required for detailed individual patient data (IPD) meta-analyses [16, 17]. The chronic pain stratification results of Aim 2 will enable the examination of baseline sample characteristics and to incorporate differences seen there when comparing across studies. When individual patient data are available, samples can be balanced by pain impact category through case-mix adjustment [33] or weighting, and subgroup analyses would be possible using methods such as IPD meta-analysis or simulation modeling. For future trials, targeting patients at the same chronic pain impact level will enhance trial efficiency by reducing patient heterogeneity, and researchers could later report on heterogeneity of treatment effect (HTE) using the groupings defined by this stratification. Finally, stratification and knowing the results of studies by chronic pain subgroup will allow interventions to be targeted to the patient subgroups where they will be the most effective.

The results of Aim 3 will produce needed information about the data quality available from the MTurk platform and provide guidance of how to ensure that quality and its limits. We will also provide information on its efficiency in time and cost for chronic pain data collection.

Whereas this study offers several benefits to chronic pain researchers, it also faces challenges. The reliability and validity of the crosswalks between different measures generated by this study will be limited by the nature of the measures and the empirical associations we observe in the datasets analyzed. If the measures tap into different constructs, linking will not solve those differences. The ISS as proposed by the NIH Task Force may not capture the appropriate dimensions of chronic pain impact, or the dimensions that best define useful subgroupings (i.e., groupings of chronic pain patients with levels of condition severity and outcomes more homogenous than seen in chronic pain patients overall). Our use of MTurk as a data collection platform for intermediate analyses may generate samples that are different enough from those seen nationally that our estimated relationships will not hold up in the final national sample analyses.

## Data Availability

This study will use data from a variety of datasets. Some of these are existing and publicly available and we will provide links to those. Others of the datasets are not publicly available due to this provision not being included in participants’ consent forms but are available from the corresponding author on reasonable request.

## Abbreviations

ACT: Assessment of Chiropractic Treatment for Low Back Pain trial
CERC: RAND Center of Excellence for the Appropriateness of Care
CFI: Comparative fit index
CLBP: Chronic low back pain
CNP: Chronic neck pain
IRT: Item response theory
ISS: Impact Stratification Score
MTurk: Amazon Mechanical Turk
NDI: Neck Disability Index
NIH: National Institutes of Health
NPS: National Pain Strategy
ODI: Oswestry Disability Index
PRO: Patient-reported outcome
PROMIS: Patient-Reported Outcomes Measurement Information System
RMDQ: Roland-Morris Disability Questionnaire
RMSEA: Root mean square error of approximation
RTF: Research task force
SRMR: Standardized root means square residual

## References

[1] Ivanova JI, Birnbaum HG, Schiller M, Kantor E, Johnstone BM, Swindle RW (2011). Real-world practice patterns, health-care utilization, and costs in patients with low back pain: the long road to guideline-concordant care. Spine J.

[2] Cramer H, Lauche R, Haller H, Dobos G (2013). A systematic review and meta-analysis of yoga for low back pain. Clin J Pain.

[3] Furlan AD, Giraldo M, Baskwill A, Irvin E, Imamura M. Massage for low-back pain. Cochrane Database Syst Rev. 2015;(9):CD001929. 10.1002/14651858.CD001929.pub3. 10.1002/14651858.CD001929.pub3 PMC8734598 26329399

[4] Henschke N, Ostelo R, van Tulder MW, Vlaeyen J, Morley S, Assendelft W, et al. Behavioural treatment for chronic low-back pain. Cochrane Database Syst Rev. 2010;7(7). 10.1002/14651858.CD002014.pub3 PMC7065591 20614428

[5] Kamper SJ, Apeldoorn A, Chiarotto A, Smeets R, Ostelo R, Guzman J (2015). Multidisciplinary biopsychosocial rehabilitation for chronic low back pain: Cochrane systematic review and meta-analysis. BMJ..

[6] Vickers AJ, Cronin AM, Maschino AC, Lewith G, MacPherson H, Foster NE (2012). Acupuncture for chronic pain: individual patient data meta-analysis. Arch Intern Med.

[7] Chou R, Atlas SJ, Stanos SP, Rosenquist RW. Nonsurgical Interventional Therapies for Low Back Pain: A Review of the Evidence for an American Pain Society Clinical Practice Guideline. [Review]. Spine. 2009;34(10):1066–77,78–93. 10.1097/BRS.0b013e3181a103b1 19363456

[8] Farabaugh RJ, Dehen MD, Hawk C (2010). Management of chronic spine-related conditions: consensus recommendations of a multidisciplinary panel. J Manip Physiol Ther.

[9] Agency for Healthcare Research and Quality. Noninvasive Nonpharmacological Treatment for Chronic Pain: A Systematic Review. Rockville, MD: Agency for Healthcare Research and Quality; 2018 June. Contract No.: AHRQ Pub. No. 18-EHC013–1-EF.

[10] Qaseem A, Wilt TJ, McLean RM, Forciea MA (2017). Noninvasive treatments for acute, subacute, and chronic low back pain: a clinical practice guideline from the American College of Physicians. Ann Intern Med.

[11] Deyo RA, Dworkin SF, Amtmann D, Andersson G, Borenstein D, Carragee E (2014). Report of the NIH task force on research standards for chronic low back pain. Pain Med.

[12] Roland M, Morris R (1983). A study of the natural history of back pain: part I: development of a reliable and sensitive measure of disability in low-back pain. Spine..

[13] Cella D. PROsetta Stone: Linking Patient-Reported Outcome Measures Bethesda, MD: National Cancer Institute; 2018 [Available from: http://www.prosettastone.org/Pages/default.aspx.

[14] Schalet BD, Rothrock NE, Hays RD, Kazis LE, Cook KF, Rutsohn JP (2015). Linking physical and mental health summary scores from the veterans RAND 12-item health survey (VR-12) to the PROMIS® Global Health scale. J Gen Intern Med.

[15] Fairbank JCT, Couper J, Davies JB, O’Brien JP (1980). The Oswestry low back pain disability questionnaire. Physiotherapy..

[16] Stewart LA, Tierney JF (2002). To IPD or not to IPD? Advantages and disadvantages of systematic reviews using individual patient data. Eval Health Prof.

[17] Stewart LA, Tierney JF, Clarke M, Higgins JPT, Green S (2011). Chapter 18: reviews of individual patient data. Cochrane handbook for systematic reviews of interventions version 510.

[18] Office of the Assistant Secretary for Health. National Pain Strategy. Washington, DC: US Department of Health and Human Services; 2016 Accessed November 9, 2017.

[19] Von Korff M, Scher AI, Helmick C, Carter-Pokras O, Dodick DW, Goulet J (2016). United States National Pain Strategy for population research: concepts, definitions, and pilot data. J Pain.

[20] Nahin RL (2015). Estimates of pain prevalence and severity in adults: United States, 2012. J Pain.

[21] Von Korff M, Ormel J, Keefe FJ, Dworkin SF (1992). Grading the severity of chronic pain. Pain..

[22] Gaskin DJ, Richard P (2012). The economic costs of pain in the United States. J Pain.

[23] Taylor-Stokes G, Lobosco S, Pike J, Sadosky AB, Ross E (2011). Relationship between patient-reported chronic low back pain severity and medication resources. Clin Ther.

[24] Elliott AM, Smith BH, Penny KI, Smith WC, Chambers WA (1999). The epidemiology of chronic pain in the community. Lancet.

[25] Engel CC, Von Korff M, Katon WJ (1996). Back pain in primary care: predictors of high health-care costs. Pain..

[26] Macfarlane GJ, Beasley M, Jones EA, Prescott GJ, Docking R, Keeley P (2012). The prevalence and management of low back pain across adulthood: results from a population-based cross-sectional study (the MUSICIAN study). Pain..

[27] Penny KI, Purves AM, Smith BH, Chambers WA, Smith WC (1999). Relationship between the chronic pain grade and measures of physical, social and psychological well-being. Pain..

[28] Smith BH, Penny KI, Purves AM, Munro C, Wilson B, Grimshaw J (1997). The chronic pain grade questionnaire: validation and reliability in postal research. Pain..

[29] Underwood MR, Barnett AG, Vickers MR (1999). Evaluation of two time-specific Back pain outcome measures. Spine..

[30] Von Korff M, Turk DC, Melzack R (2011). Assessment of chronic pain in epidemiological and health services research: empirical bases and new directions. Handbook of Pain Assessment.

[31] Herman PM, Broten N, Lavelle TA, Sorbero ME, Coulter ID (2019). Exploring the prevalence and characteristics of high-impact chronic pain across chronic low-Back pain study samples. Spine Journal.

[32] Herman PM, Broten N, Lavelle TA, Sorbero ME, Coulter ID (2019). Healthcare costs and opioid use associated with high-impact chronic spinal pain in the United States. Spine..

[33] O'Malley AJ, Zaslavsky AM, Elliott MN, Zaborski L, Cleary PD (2005). Case-Mix Adjustment of the CAHPS® Hospital Survey. Health Serv Res.

[34] Kent DM, Rothwell PM, Ioannidis JP, Altman DG, Hayward RA (2010). Assessing and reporting heterogeneity in treatment effects in clinical trials: a proposal. Trials..

[35] Deyo RA, Ramsey K, Buckley DI, Michaels L, Kobus A, Eckstrom E (2015). Performance of a patient reported outcomes measurement information system (PROMIS) short form in older adults with chronic musculoskeletal pain. Pain Med.

[36] Ipsos. Be Sure with KnowledgePanel New York: Ipsos; 2019 [Available from: https://www.ipsos.com/en-us/solution/knowledgepanel.

[37] Samuel A. Amazon Mechanical Turk has Reinvented Research. JSTOR. 2018;May 15, 2019(https://daily.jstor.org/amazons-mechanical-turk-has-reinvented-research/).

[38] Hitlin P. Research in the Crowdsourcing Age, a Case Study Washington, DC: Pew Research Center; 2016 [updated July. Available from: http://www.pewinternet.org/2016/07/11/research-in-the-crowdsourcing-age-a-case-study/.

[39] Cook C (2011). Grassroots clinical research using crowdsourcing. J Man Manip Ther.

[40] Ranard BL, Ha YP, Meisel ZF, Asch DA, Hill SS, Becker LB (2014). Crowdsourcing—harnessing the masses to advance health and medicine, a systematic review. J Gen Intern Med.

[41] Shapiro DN, Chandler J, Mueller PA (2013). Using mechanical Turk to study clinical populations. Clin Psychol Sci.

[42] Sheehan KB (2018). Crowdsourcing research: data collection with Amazon’s mechanical Turk. Commun Monogr.

[43] Antoun C, Zhang C, Conrad FG, Schober MF (2016). Comparisons of online recruitment strategies for convenience samples: craigslist, Google AdWords, Facebook, and Amazon mechanical Turk. Field Methods.

[44] Coulter ID, Herman PM, Ryan GW, Hays RD, Hilton LG, Whitley MD (2019). Researching the appropriateness of care in the complementary and integrative health professions: part 1. J Manip Physiol Ther.

[45] Coulter ID, Herman PM, Ryan GW, Hays RD, Hilton LJ, Team CERC (2019). The challenge of determining appropriate Care in the era of patient-centered care and rising health care costs. J Health Serv Res Policy.

[46] Herman PM, Kommareddi M, Sorbero ME, Rutter CM, Hays RD, Hilton LG (2018). Characteristics of chiropractic patients being treated for chronic low back and chronic neck pain. J Manip Physiol Ther.

[47] Goertz CM, Long CR, Vining RD, Pohlman KA, Kane B, Corber L (2016). Assessment of chiropractic treatment for active duty, US military personnel with low back pain: study protocol for a randomized controlled trial. Trials..

[48] Goertz CM, Long CR, Vining RD, Pohlman KA, Walter J, Coulter I (2018). Effect of usual medical care plus chiropractic care vs usual medical care alone on pain and disability among US service members with low back pain: a comparative effectiveness clinical trial. JAMA Network Open.

[49] Hilton LG, Azzam T. Crowdsourcing qualitative thematic analysis. American Journal of Evaluation. 2019;ePub April 9:1–15.

[50] PROMIS 1 Wave 1. Harvard University. 2015 [Cited July 8, 2020]. Available from: 10.7910/DVN/0NGAKG.

[51] PROMIS 1 American Chronic Pain Association (ACPA) Supplement [Internet]. Harvard University. 2016 [Cited July 8, 2020]. Available from: 10.7910/DVN/5JAACI.

[52] PROMIS 1 Profiles-HUI data [Internet]. Harvard University. 2017 [Cited July 8, 2020]. Available from: 10.7910/DVN/P7UKWR.

[53] Chapman JR, Norvell DC, Hermsmeyer JT, Bransford RJ, DeVine J, McGirt MJ (2011). Evaluating common outcomes for measuring treatment success for chronic low back pain. Spine..

[54] Tsang A, Von Korff M, Lee S, Alonso J, Karam E, Angermeyer MC (2008). Common chronic pain conditions in developed and developing countries: gender and age differences and comorbidity with depression-anxiety disorders. J Pain.

[55] Johannes CB, Le TK, Zhou X, Johnston JA, Dworkin RH (2010). The prevalence of chronic pain in United States adults: results of an internet-based survey. J Pain.

[56] Vernon H (2008). The neck disability index: state-of-the-art, 1991-2008. J Manip Physiol Ther.

[57] MacDermid JC, Walton DM, Côté P, Santaguida PL, Gross A, Carlesso L (2013). Suppl 4: Use of Outcome Measures in Managing Neck Pain: An International Multidisciplinary Survey. Open Orthopaedics J.

[58] Dorans NJ (2007). Linking scores from multiple health outcome instruments. Qual Life Res.

[59] Hu L, Bentler PM (1999). Cutoff criteria for fit indexes in covariance structure analysis: conventional criteria versus new alternatives. Struct Equ Model Multidiscip J.

[60] Cook KF, Kallen MA, Amtmann D (2009). Having a fit: impact of number of items and distribution of data on traditional criteria for assessing IRT’s unidimensionality assumption. Qual Life Res.

[61] Xia Y, Yang Y. RMSEA, CFI, and TLI in structural equation modeling with ordered categorical data: the story they tell depends on the estimation methods. Behav Res Methods. 2018:1–20. 10.3758/s13428-018-1055-2 29869222

[62] Hays RD, Madans J, Miller K, Maitland A, Willis G (2011). Response 1 to Reeve’s chapter: applying item response theory for questionnaire evaluation. Question evaluation methods: contributing to the science of data quality.

[63] Samejima F. Estimation of latent ability using a response pattern of graded scores. Richmond, VA: Psychometric Society; 1969. Report No.: Psychometric Monograph No. 17.

[64] Rasch G (1960). Probabilistic models for some intelligence and attainment tests.

[65] Edelen MO, Stucky BD, Sherbourne C, Eberhart N, Lara M (2014). Correspondence between the RAND–negative impact of asthma on quality of life item Bank and the Marks asthma quality of life questionnaire. Clin Ther.

[66] Fayers PM, Hays RD (2014). Should linking replace regression when mapping from profile-based measures to preference-based measures?. Value Health.

[67] Lapin BR, Kinzy TG, Thompson NR, Krishnaney A, Katzan IL. Accuracy of linking VR-12 and PROMIS Global Health scores in clinical practice. Value Health. 2018. 10.1016/j.jval.2018.03.011 30314624

[68] Mandrekar JN (2010). Receiver operating characteristic curve in diagnostic test assessment. J Thorac Oncol.

[69] Edwards RR, Cahalan C, Mensing G, Smith M, Haythornthwaite JA (2011). Pain, catastrophizing, and depression in the rheumatic diseases. Nat Rev Rheumatol.

[70] Arnow BA, Blasey CM, Constantino MJ, Robinson R, Hunkeler E, Lee J (2011). Catastrophizing, depression and pain-related disability. Gen Hosp Psychiatry.

[71] Bishop FL, Yardley L, Prescott P, Cooper C, Little P, Lewith GT (2015). Psychological covariates of longitudinal changes in back-related disability in patients undergoing acupuncture. Clin J Pain.

[72] Turner JA, Holtzman S, Mancl L (2007). Mediators, moderators, and predictors of therapeutic change in cognitive–behavioral therapy for chronic pain. Pain..

[73] Burns J, Glenn B, Bruehl S, Harden R, Lofland K (2003). Cognitive factors influence outcome following multidisciplinary chronic pain treatment: a replication and extension of a cross-lagged panel analysis. Behav Res Ther.

[74] Burns JW, Kubilus A, Bruehl S, Harden RN, Lofland K (2003). Do changes in cognitive factors influence outcome following multidisciplinary treatment for chronic pain? A cross-lagged panel analysis. J Consult Clin Psychol.

[75] Jensen MP, Turner JA, Romano JM (2001). Changes in beliefs, catastrophizing, and coping are associated with improvement in multidisciplinary pain treatment. J Consult Clin Psychol.

[76] Leeuw M, Goossens ME, van Breukelen GJ, de Jong JR, Heuts PH, Smeets RJ (2008). Exposure in vivo versus operant graded activity in chronic low back pain patients: results of a randomized controlled trial. Pain..

[77] Smeets RJ, Vlaeyen JW, Kester AD, Knottnerus JA (2006). Reduction of pain catastrophizing mediates the outcome of both physical and cognitive-behavioral treatment in chronic low back pain. J Pain.

[78] Abshire M, Dinglas VD, Cajita MIA, Eakin MN, Needham DM, Himmelfarb CD (2017). Participant retention practices in longitudinal clinical research studies with high retention rates. BMC Med Res Methodol.

[79] Litman L, Robinson J, Abberbock T (2017). TurkPrime. Com: a versatile crowdsourcing data acquisition platform for the behavioral sciences. Behav Res Methods.

[80] Hilton L (2018). Advancing democratic evaluation: using crowdsourcing to include and engage program participants.

[81] Siegel JT, Navarro MA, Thomson AL (2015). The impact of overtly listing eligibility requirements on MTurk: an investigation involving organ donation, recruitment scripts, and feelings of elevation. Soc Sci Med.

[82] Brown TA. Confirmatory factor analysis for applied research. New York: Guilford; 2006.

[83] Reeve B, Fayers P (2005). Applying item response theory modelling for evaluating questionnaire item and scale properties. Assessing Quality of Life in Clinical Trials.

